# Longitudinal resident coaching in the outpatient setting: A novel intervention to improve ambulatory consultation skills

**DOI:** 10.1007/s40037-020-00573-5

**Published:** 2020-03-30

**Authors:** Ryan Graddy, Stasia S. Reynolds, Scott M. Wright

**Affiliations:** 1 Division of Addiction Medicine, Department of Medicine, Johns Hopkins Bayview Medical Center, Johns Hopkins University School of Medicine, Baltimore, MD USA; 2 Division of General Internal Medicine, Department of Medicine, Johns Hopkins Bayview Medical Center, Johns Hopkins University School of Medicine, Baltimore, MD USA

**Keywords:** Coaching, Graduate medical education, Direct observation, Deliberate practice

## Abstract

**Background:**

Direct observation with feedback to learners should be a mainstay in resident education, yet it is infrequently done and its impact on consultation skills has rarely been assessed.

**Approach:**

This project presents the framework and implementation of a longitudinal low-frequency, high-intensity direct observation and coaching intervention, and elaborates on insights learned. Internal medicine interns at one residency training program were randomized to an ambulatory coaching intervention or usual precepting. Over one year, coached interns had three complete primary care visits directly observed by a faculty clinician-coach who provided feedback informed by a behavior checklist. Immediately after each of the coached patient encounters, interns completed a structured self-assessment and coaches led a 30-minute feedback session informed by intern self-reflection and checklist items. Interns with usual precepting had two mini-CEX observations over the course of the year without other formal direct observation in the ambulatory setting.

**Evaluation:**

As part of the post-intervention assessment, senior faculty members blinded to intervention and control group assignments evaluated videotaped encounters. Coached interns completed an average of 21/23 behaviors from the checklist, while interns from the control group completed 18 (*p* < 0.05). The median overall grade for coached interns was B+, compared to B−/C+ for controls (*p* < 0.05).

**Reflection:**

Coaching interns longitudinally using a behavior checklist is feasible and associated with improved consultation performance. Direct observation of complete clinical encounters followed by systematic coaching is educationally valuable, but time and resource intensive.

## Background and need for innovation

There is widespread agreement that direct observation and feedback on clinical practice is critical for professional growth during residency education; this formative assessment is believed to be invaluable [1, 2]. Residency training has the potential for development of rich consultation skills—with repeated, intense practice alongside peers and under the supervision of committed educators. Despite these opportunities, the vast majority of resident clinical practice is unobserved by anyone other than the patient. Usual precepting of resident physicians is of short duration, often haphazard, and can involve fragmented supervision by faculty preceptors. Because faculty cannot offer constructive feedback on behaviors they have not seen, residents often rely on self-assessments which are commonly inaccurate [3, 4]. Time constraints, the absence of structure to accommodate direct observation, and lack of financial compensation have all been cited as barriers to incorporating direct observation regularly into graduate medical education [5].

Coaching paradigms place emphasis on self-assessment, reflection-on-practice, and thoughtfully stimulated feedback immediately following direct observation [6–8]. While focused, episodic direct observation has been shown to improve some procedural techniques in surgical training [9], its impact on observed consultation performance—particularly longitudinally—has not previously been studied in outpatient settings.

## Goal of innovation

We set out to develop and evaluate a longitudinal direct observation and coaching intervention designed to improve outpatient consultation skills among internal medicine interns. Here, consultation skills refers to the ingredients of an outpatient primary care encounter including professionalism, verbal and non-verbal communication, medical knowledge, diagnostic acumen, use of electronic medical records, and negotiation of the healthcare system. In this article, we discuss the conceptual framework and implementation of our intervention, present outcomes data from our pilot study, and share details about the successes and pitfalls that can inform other educators and training programs who may be considering similar educational methods.

## Development and implementation of innovation

This direct observation intervention was grounded in the coaching paradigm, a model that places an emphasis on reflection-on-practice, self-assessment, and direct observation by a skilled ‘coach’ who provides detailed, specific, and timely feedback [6–8]. Using this model in graduate medical education, residents engage in self-assessment of their performance prior to receiving feedback from trusted faculty educators; this practice allows trainees to develop refined self-assessment techniques and to enhance their consultation skills [10]. Clinical coaching requires repeated direct observation and a longitudinal relationship between the learner and the experienced guide [11].

The intervention was implemented with interns in the Johns Hopkins Bayview Internal Medicine Residency in Baltimore, MD, over the course of the 2017–18 academic year. At the beginning of the year, half of the interns (8 of the 16 trainees) were randomly selected (using random.org) to the intervention group where they would be longitudinally coached by one of two faculty preceptors with extensive coaching and precepting experience. The four male interns (25%) were equally distributed between the two study groups. Over the course of 12 months, each coach performed three complete outpatient visit observations with each of their learners. During these patient encounters, faculty remained in the room for the entirety of a patient visit; a 23-item consultation behavioral checklist, adapted from previous work [12], was used to focus the observations. Immediately following each encounter, there was a 30-minute coaching and debriefing session. First, the intern would fill out a self-assessment that included the behavioral checklist. Next, the intern would be asked to reflect on his/her performance, guided by insightful questioning by the coach. Finally, the coach would deliver specific, detailed feedback about the intern’s performance. These formative feedback sessions were used to capitalize on longitudinal coaching relationships between faculty and interns through the creation of specific goal-setting for future outpatient encounters, as well as reviewing progress toward previously articulated goals.

The eight interns who did not receive any targeted intervention in coaching or direct observation received the standard outpatient precepting that was offered to all residents. This includes twice annual partially observed encounters by faculty using a mini-CEX format [13], which was also continued for interns in the intervention group.

## Evaluation of the innovation

At the end of the academic year, 15/16 interns had a complete patient visit in the practice videotaped. Encounters that were videotaped were selected as a convenience sample on days in June 2018 when a study team member (SR) was available to do so. One intern in the control group declined to be videotaped for the project, all others consented. Interns were asked to deliver care as per usual and to disregard the taping; no guidance was given to the interns about who would view the tapes or how they would be scored.

Seven experienced general internal medicine and geriatrics faculty members (average 26 years post-residency clinical practice) with no direct involvement in the coaching portion of the project were recruited to independently evaluate videotaped intern consultation performances. These clinician-educators received an email introduction to the project and were asked if they would be willing to volunteer as video raters; they were not compensated for their time. They were blinded to intervention and control group interns and were given brief email training on use of the same 23-item checklist used by coaching faculty in the intervention group. Additionally, raters were asked to assign a letter grade (A–F) to each video based on their evaluation of the intern’s consultation performance, independent of the checklist and instead based on their overall impression. Grades were transitioned from letters to numbers for statistical analysis where F = 0 and A+ = 12. Each video was viewed by two raters. Discrepant yes/no responses were reconciled to ‘yes,’ where one rater’s identification of a completed behavior was deemed accurate over another’s reported omission of the behavior (this occurred in 19% of items and equally in both groups). The a priori primary outcome of the analysis for this study was the number of items observed on the video as per the assessment of the raters. We hypothesized that coached interns would perform a higher proportion of checklist behaviors and receive higher grades than interns in the control group. The proportions of each behavior performed were compared using chi-squared tests, and differences in the two groups’ overall performance of behaviors on the checklist and grades were assessed with two-tailed Mann-Whitney U tests with α = 0.05. This study was reviewed and deemed exempt by the Johns Hopkins University School of Medicine Institutional Review Board.

**Fig. 1:**
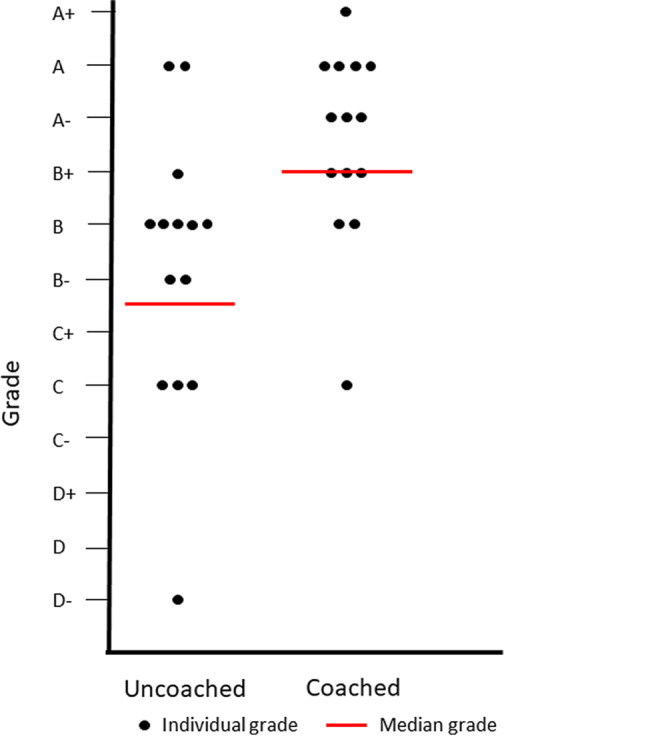
Intern grade distribution

On the videotaped encounters, eleven of the 23 behaviors were executed with greater frequency among interns who had been coached, and 2/23 were performed more often among the controls. No individual checklist items were performed statistically significantly more often by either group (all *p* > 0.05, see Tab. 1). Overall, coached interns were judged to complete a median of 19/23 behaviors, while uncoached interns completed 16 (Mann-Whitney *U—p* < 0.05 two-tailed). The median overall grade for coached interns was B+, compared with B−/C+ for the controls (Mann-Whitney *U—p* < 0.05 two-tailed). The distribution of grades across groups is shown in the Fig. 1.

**Table 1:** Intern completion of checklist behaviors as assessed by experienced senior clinicians at end of academic year

Checklist item	Coached	Control	*p* value
	*N* = 8 (% complete)	*N* = 7 (% complete)	
Is fully present during encounter	8 (100)	7 (100)	1.00
Listens attentively	8 (100)	7 (100)	1.00
Avoids medical jargon	8 (100)	7 (100)	1.00
Positions self to facilitate communication	8 (100)	7 (100)	1.00
Gives undivided attention to patient before/after charting	8 (100)	7 (100)	1.00
Employs adequate eye contact when using EMR	8 (100)	7 (100)	1.00
Obtains adequate information to arrive at diagnosis/plan	8 (100)	7 (100)	1.00
Interprets data and formulates plan appropriate for problem	8 (100)	7 (100)	1.00
Creates comprehensive management plan for patient	8 (100)	7 (100)	1.00
Enables patient to understand comprehensive management plan	8 (100)	7 (100)	1.00
Describes evidence base to treat patient	5 (63)	5 (71)	0.71
Closes visit with open-ended question	6 (75)	4 (57)	0.46
Completes appropriate physical exam	7 (88)	7 (100)	0.33
Asks open-ended questions	8 (100)	6 (86)	0.27
Collaborates with patient using EMR	8 (100)	6 (86)	0.27
Manages visit time appropriately	8 (100)	6 (86)	0.27
Counsels on behavioral change	6 (75)	3 (43)	0.21
Uses appropriate physical exam technique	6 (75)	3 (43)	0.21
Outlines reasons to re-contact	6 (75)	3 (43)	0.21
Demonstrates ability to prioritize multiple issues during visit	7 (88)	4 (57)	0.19
Addresses social/financial concerns	7 (88)	4 (57)	0.19
Asks thoughtful questions	8 (100)	5 (71)	0.10
Assesses understanding (teach back)	6 (75)	2 (29)	0.07

## Critical reflection

This longitudinal, structured coaching intervention in the ambulatory setting demonstrates promise as a model for increasing the frequency of high quality, focused direct observation of resident encounters with patients. Interns experienced the value of reflection on practice, self-assessment, and collaborating with a coach. This intervention was associated with better overall consultation performance and completion of more checklist items compared with usual ambulatory precepting.

Previous direct observation and coaching interventions in the outpatient setting have demonstrated acceptability among residents and faculty, improved resident confidence in faculty feedback, and better identification of blind spots in resident self-assessment [11, 13, 14]. This paper adds to this sparse literature by describing how this is grounded in a conceptual framework and providing some evidence for its educational value in changing behaviors.

In the course of formulating and executing the intervention, we learned a great deal. First, we found that directly observing the entirety of outpatient consultation encounters using a detailed checklist provided substantially more information than traditional precepting or sporadic mini-CEXes. Witnessing a whole visit allowed coaches to evaluate interns’ ability to prioritize multiple issues during a visit and manage time, skills that are incompletely captured in partial evaluations yet are important to understand. Further, the checklist provided more specific details than the broad categories included in the mini-CEX checklist; it breaks down outpatient consultation skills into discrete behaviors. Previous studies suggest that feedback from mini-CEXes is often felt to be non-specific and of limited value [15, 16]. Formative assessment of discrete, actionable behaviors is an important means of creating consistency in evaluations and for providing specific feedback. The longitudinal nature of our intervention led to the development of trusting coach-coachee relationships in which feedback became more focused and targeted over time. Coaching relationships in other disciplines, including music and sports, depend on longitudinal exposure in which feedback is more easily tailored to the individual over repeated sessions and evolves over time [17]. In medical student training, learners perceive preceptor assessment and feedback as more authentic when observed consistently by a single faculty expert [18]. This longer and more consistent coaching contact with residents may be difficult to schedule; in addition, this is both time and resource intensive. The optimal number of coached patient encounters and duration of coaching relationship are unknown. Finally, our project benefited significantly from institutional support to pursue coaching; both faculty members serving as coaches had built-in flexibility to their academic schedules to pursue projects to improve the quality of ambulatory resident education. Previous work has shown that the quality of clinical teaching is linked to the amount of protected time in academic settings [19, 20].

Several limitations of this study should be considered. First, this project was conducted at a single clinical site in a single internal medicine residency program. Second, the number of interns in our training program is fairly small, so there were few trainees in the intervention and control groups. With a small n, it is possible that some results may be due to chance. Given the frequent interactions of interns in the control and coached groups in and out of clinic, it is also possible that coached interns shared some pearls of wisdom learned through coaching with the other interns. Third, the faculty raters who reviewed videotaped encounters may not have interpreted items exactly as intended. We sought to minimize the problem by providing them with brief training in how to complete the checklist; as such, we gave learners credit for performance of a behavior if either independent observer endorsed completion of the behavior, because it may have been difficult for observers to track all checklist items. Fourth, the checklist has not been validated as a summative scoring rubric. Finally, the coached group’s familiarity with the checklist may have influenced their ability to perform better on the videotaped encounter.

Further evaluations of the utility and viability of this direct observation and coaching strategy in other settings will help to determine its broader applicability. Future steps may include validation of the checklist tool and application to other resident practices to assess both feasibility and impact on trainees’ consultation skills. Additional work to evaluate the impact of different doses of coaching on learner consultation performance may also assist in the development of the best and most efficient models for outpatient coaching.

In conclusion, this pilot found that an innovative longitudinal outpatient direct observation and coaching intervention for interns was associated with improved consultation performance compared with usual precepting in the ambulatory setting.
